# Editorial: Reviews in genitourinary oncology

**DOI:** 10.3389/fonc.2023.1196769

**Published:** 2023-04-13

**Authors:** Stefano Arcangeli, Andrea Lancia, Gianluca Ingrosso, Luca Triggiani, Anna Wilkins

**Affiliations:** ^1^ Department of Radiation Oncology, University of Milan, Bicocca, Italy; ^2^ Department of Radiation Oncology, San Matteo Hospital Foundation Istituto di Ricovero e Cura a Carattere Scientifico (IRCCS), Pavia, Italy; ^3^ Radiation Oncology Section, University of Perugia, Perugia, Italy; ^4^ Department of Radiation Oncology, University of Brescia, Brescia, Italy; ^5^ Division of Radiotherapy and Imaging, Institute of Cancer Research, London, United Kingdom

**Keywords:** GU malignancies, biomarkers, clinical research (CRE), tumor biology, precision medicine

GU malignancies represent approximately one fourth of all solid tumors and over the past 20 years there have been huge improvements due to a rapid evolution in diagnostic modalities, with the emergence of novel biomarkers and clinical validation of new diagnostic tools, and to a broadening of therapeutic options. The understanding of the molecular interplay within the tumor microenvironment has resulted in an improved drug design and discovery, with the rise of novel agents, including second-generation anti-androgens, radioactive molecules, PARP inhibitors, checkpoint inhibitors and targeted therapy, each enabling to implement a precision medicine approach. In parallel, dramatic changes have also occurred in the field of radiation oncology, due to its ability to achieve a higher conformality around the target while reducing the dose to the surrounding healthy tissues, as well as in urological surgery, that has safely incorporated minimally invasive endoscopic techniques.

This Research Topic is aimed at widening the knowledges in various aspects of GU oncology, emphasizing interdisciplinary contributions. The issue currently includes 18 reviews/metanalyses mainly covering renal, bladder, and prostate cancers from different perspectives including basic science, genomic, clinical research, and translational research. All contributions to this Research Topic focus on one or more of the aforementioned research areas.

One of the hot topics deals with the relevant advances and main changes in the imaging in GU oncology. Liu et al. evaluated the detection rate of fluoro-prostate-specific membrane antigen (18F-PSMA-1007) PET/CT in patients with different serum PSA levels in the setting of both primary staging and biochemically recurrent prostate cancer. They found that the detection rate of 18F-PSMA-1007 PET/CT was slightly higher in primary prostate tumors than in biochemical recurrence, and that it is improved with increasing serum PSA levels. Tian et al. assessed a multiparametric MRI clear cell likelihood score algorithm for the classification of small renal masses: for all cT1 renal masses, the pooled sensitivity and specificity were 0.80 (95% CI 0.74–0.85) and 0.76 (95% CI 0.67–0.83), respectively, showing a moderate to high accuracy for identifying clear cell RCC from other RCC subtypes, and with a moderate inter-reader agreement.

Biomarkers have become a significant focus of research, and namely on how they can help predict response to systemic therapy, identify treatment resistance, and tumors’ immunogenity. Xie et al. investigated the diagnostic, clinicopathological, and prognostic utility of circRNAs in prostate cancer: the aggregated data from their metanalysis revealed an AUC of 0.81, with a sensitivity of 0.82 and a specificity of 0.62 for diagnostic value, indicating that circRNAs could be employed as diagnostic biomarkers for prostate cancer, and that aberrant circRNA expression was strongly linked to poor overall survival in terms of prognostic value. Rizzo et al. demonstrated a robust rationale for the use of IL-8 as a potential prognostic and predictive biomarker in the use of ICIs and TKI in mRCC, which has the potential to customize the treatment for each individual patient, although confirmatory studies are needed. Jiang et al. focused on the role of tumor-derived exosomes in clear cell RCC metastasis, drug resistance and diagnosis, and highlighted the potential to act as biological markers and meaningful targets for early diagnosis and monitoring of disease at once. Burley et al. explored the basics of cancer-associated fibroblasts (CAFs) biology in bladder cancer and identified key therapeutic challenges associated with CAFs, such as the lack of specific CAF markers, the paucity of research into bladder-specific CAFs and their relationship with inferior responses to radical radiotherapy, and also the opportunities of being employed as single agents and in combination with existing therapies. Wu et al. reported on the recent findings on lactate dehydrogenase C4 (LDH-C4) and highlighted that not only it can be employed as an important parameter in evaluating semen quality and male reproductive function but has the potential to provide new clues for the early diagnosis of testicular tumors.

Immunotherapy, that has historically been centered on systemic cytokines for the treatment of metastatic kidney cancer, or the Bacillus Calmette–Guérin vaccine for non-metastatic bladder cancer, has enormously evolved in the past decade, especially in the field of immune checkpoint inhibition, to a point that immune checkpoint inhibitors (ICIs) are now used extensively in the treatment of kidney and bladder cancers. Wang et al. summarized the recent studies that looked at the interaction networks of B cells with other cells, discussed the role of B cells in RCC development and progression, and assessed their impact on RCC immunotherapy, while Raghubar et al. examined the mechanisms behind the transition of proximal tubular epithelial cells (PTEC) in clear cell RCC development, and the interactions that may limit the response to targeted immune therapy, finally concluding that stromal cells are key drivers in recurrent and locally invasive clear cell RCC.

The uro-oncologic treatment’s landscape has seen a tremendous growth with major advancements in prostate, bladder, and urothelial cancers due to a better understanding of tumour biology and the underlying genetic and molecular alterations. Zhu et al. summarized the current data and addressed whether neoadjuvant (NAC) or adiuvant (AC) chemotherapy is effective for variant histologies bladder cancers. In general, they found that favorable OS and CSS observed in patients with frequent histologies who receive NAC or AC are confirmed in those with variant histologies. Interestingly however, a subgroup analysis revealed that NAC independently improved OS in sarcomatoid and neuroendocrine tumors but not in squamous histology. With the development of molecular research, a variety of biomarkers are expected to predict the response to cisplatin-based chemotherapy, thus driving the use of NAC/AC in the future, as also pointed out by Roviello et al., who scrutinized the role of neoadjuvant therapy in muscle invasive bladder cancer (MIBC), highlighting recent advances that can change the clinical practice, and concluded that molecular signatures have the potential for reshaping the selection for tailored treatment and disease monitoring. Zheng et al. show that erdafitinib, a pan-FGFR inhibitor, resulted in a higher objective response rate (0.38 versus 0.10) and lower progressive disease rate (0.26 versus 0.68) in urothelial carcinoma patients compared to those with other solid tumor patients, and that the drug was more effective in presence of fibroblast growth factor receptor (FGFR) alteration, particularly when a specific FGFR alteration (FGFR3-TACC3) was observed. Kloskowski et al. shed light on the potential therapeutic activity of Quinolones, a broad-spectrum antibiotics frequently prescribed by urologists due to their higher accumulation in urine and prostate tissue than in serum, and speculated that the use of modified quinolones in combination with other chemotherapeutics can enable toxic effects at lower drug doses in bladder cancer treatment.

A significant progress has also occurred in the surgical treatment of bladder cancer, mainly due to the implementation of robotic surgery, as emphasized by Long et al. who analyzed the difference in efficacy between robotic-assisted radical cystectomy (RARC) and laparoscopic radical cystectomy (LRC) in bladder cancer and found that the former one is a safe and effective treatment with reduced surgical blood loss and postoperative complications compared to LRC.

Remarkable achievements in the recent years have revolutionized the management of advanced prostate cancer with the opportunity of customizing treatment to the specific cancer and the individual patient. Wolf et al. have discussed the emerging knowledge about the role of Prostate Cancer Stem Cells (PCSCs) as a potential therapeutic target, although a selective and effective targeting of PCSCs remains challenging at this stage, and efforts are needed to improve the characterization of PCSCs using (single-cell) genomics and proteomics. Li et al. explored the current landscape in the management of metastatic hormone-sensitive prostate cancer (mHSPC) following the development of several novel agents and the combination of different therapeutic strategies. Chen et al. provided an overview of the research on bone metastases in prostate cancer based on a bibliometric analysis covering the past 22 years, and in particular showed that the latest research focused on the tumor microenvironment and biomarkers with the aim of exploring the mechanism and the therapeutic targets of bone metastases.

Finally, Camero et al. elucidated the mechanisms of the radioresistance of rhabdomyosarcoma (RMS), the most common soft tissue sarcoma in children, frequently accounting the genitourinary tract. This knowledge paves the way to combined therapeutic approaches that can radiosensitize cancer cells to finally ameliorate the overall survival of patients with RMS, especially for the most aggressive subtypes.

In conclusion, this Research Topic displayed exciting developments in the diagnosis and treatment of GU malignancies incorporating novel mechanisms, biomarkers for selection of targeted therapy, and innovative treatment approaches. Advances in the management of patients with bladder cancer, prostate cancer, and renal cell carcinoma can be expected from these efforts.

## Author contributions

All authors listed have made a substantial, direct, and intellectual contribution to the work and approved it for publication.

